# Severe Hypernatremia During Continuous Hemofiltration in an End-Stage Renal Disease (ESRD) Patient From the Kingdom of Saudi Arabia: A Case Report and Updated Review

**DOI:** 10.7759/cureus.96390

**Published:** 2025-11-08

**Authors:** Fahad S Alrashidi

**Affiliations:** 1 Internal Medicine/Nephrology, Ad Diriyah Hospital, Riyadh Third Health Cluster, Riyadh, SAU

**Keywords:** clinical nephrology, continuous renal replacement therapy (crrt), internal medicine, medical intensive care unit, on dialysis, sodium disorder

## Abstract

We report a case of a 74-year-old female with end-stage renal disease (ESRD) on maintenance hemodialysis who was admitted with community-acquired pneumonia and decreased level of consciousness. She required intubation and was transferred to the intensive care unit, where continuous renal replacement therapy (CRRT) was initiated. Her initial serum sodium was markedly elevated at 154 mmol/L. Careful adjustment of dialysate and replacement fluid sodium concentrations allowed gradual correction, avoiding cerebral edema or osmotic demyelination. This case highlights the importance of individualized CRRT prescription and close biochemical monitoring to achieve safe sodium correction in critically ill patients.

## Introduction

Hypernatremia affects approximately 1-4% of hospitalized patients and up to 10% of those in the intensive care unit, and it is consistently associated with increased mortality, particularly in the elderly and in patients with sepsis or renal failure. In critically ill patients receiving renal replacement therapy, dysnatremias are frequent because of impaired free-water handling, variable nutritional intake, and exposure to high-sodium dialysate and replacement solutions. Continuous renal replacement therapy (CRRT) offers the advantage of gradual correction of chronic dysnatremia; however, inappropriate sodium prescription may lead either to overly rapid changes in tonicity or to persistent hypernatremia despite treatment. Only a limited number of reports have described practical, bedside strategies to individualize CRRT sodium prescriptions, and data from the Middle East are scarce [1-4].

We present a case of severe hypernatremia in a patient with ESRD treated with CRRT, followed by an updated, practical review of management strategies focusing on sodium adjustment during CRRT.

## Case presentation

A 74-year-old woman with end-stage renal disease on thrice-weekly hemodialysis presented with cough, fever, and progressive drowsiness. On arrival, she was febrile and hypotensive, with a blood pressure of 106/70 mmHg, heart rate 97 beats/min, respiratory rate 23 breaths/min, and oxygen saturation of 92% on 8 L/min via nasal cannula. Her Glasgow Coma Scale (GCS) score was 3/15, with impaired airway-protective reflexes. Arterial blood gas analysis showed pH 7.32, PaCO₂ 30 mmHg, PaO₂ 56 mmHg, HCO₃⁻ 15 mmol/L, and lactate 3.5 mmol/L, consistent with hypoxemic respiratory failure and sepsis. She was therefore intubated primarily to secure the airway and to improve oxygenation and ventilation rather than for confusion alone.

Initial laboratory investigations showed a serum sodium of 154 mmol/L, potassium 4.2 mmol/L, blood urea nitrogen 34 mmol/L, and creatinine 890 µmol/L. A summary of the patient’s laboratory findings and corresponding normal ranges is provided in Table 1.

**Table 1:** Laboratory findings in this patient upon admission, compared to normal reference ranges BUN: blood urea nitrogen

Parameter	Patient value	Normal range
Sodium (mmol/L)	154	135–145
Potassium (mmol/L)	4.2	3.5–5.0
BUN (mmol/L)	34	2.5–7.1
Creatinine (µmol/L)	890	45–110
Glucose (mmol/L)	6.2	3.9–7.8
Calcium (mmol/L)	2.1	2.1–2.6
Chloride (mmol/L)	115	98–107
Bicarbonate (mmol/L)	22	22–29

Initial management included broad-spectrum antibiotics for community-acquired pneumonia, cautious crystalloid resuscitation, and vasopressor support as needed. Given the combination of ESRD, hemodynamic instability, and severe hypernatremia, CRRT was selected over intermittent hemodialysis to allow slow, controlled sodium correction.

CRRT was initiated using a citrate anticoagulation protocol. CRRT was delivered in a continuous veno-venous hemofiltration/hemodiafiltration (CVVHDF) mode with a blood flow of approximately 200 mL/min and an effluent dose of about 25 mL/kg/h. Dialysate flow was set at 1,000 mL/h and replacement fluid at 800 mL/h, with 25% of the replacement given pre-filter and the remainder post-filter. Net ultrafiltration was initially prescribed at 0-50 mL/h and subsequently adjusted according to hemodynamic tolerance and cumulative fluid balance.

The decision to add 100 mL of 3% NaCl (≈51 mmol of sodium) to each 5-L bag was based on published adjustment tables (Table 2) and simple mass-balance calculations aiming for a circuit sodium concentration close to the patient’s initial serum sodium (≈150 mmol/L). This approach minimized the immediate sodium gradient between blood and dialysate/replacement fluid, thereby reducing the risk of overly rapid tonicity shifts.

**Table 2:** Effect of adding 3% NaCl to a 5-L bag of replacement fluid (baseline Na⁺ 140 mmol/L) Adapted from Baeg et al. [4]

Volume of 3% NaCl added (mL)	Approx. Na⁺ added (mmol)	Final Na⁺ concentration (mmol/L)
0	0	140
50	25.7	145
100	51.3	150
150	76.9	155
200	102.6	160

Given the marked hypernatremia, the therapeutic goal was to reduce sodium gradually without exceeding 10 mmol/L per 24 hours. The sodium concentration of the dialysate and replacement fluid was customized by adding 3% NaCl to the standard 5-L solution (baseline Na⁺ 140 mmol/L) following published CRRT adjustment tables. Specifically, 100 mL of 3% NaCl (≈51 mmol Na⁺) was added per bag, resulting in an effective circuit sodium of approximately 150 mmol/L. Serial sodium levels were monitored every four to six hours, and the mixture was adjusted stepwise. After 24 hours, serum sodium decreased to 149 mmol/L, and by 48 hours it reached 145 mmol/L, remaining within the safe correction limit. The patient’s neurological status improved with no evidence of osmotic demyelination or cerebral edema. She was successfully extubated after four days and subsequently resumed intermittent hemodialysis.

This case illustrates the practicality of individualized CRRT prescription for dysnatremia, emphasizing the importance of gradual sodium correction and frequent biochemical monitoring.

## Discussion

Severe hypernatremia in dialysis patients most commonly reflects a mismatch between free-water intake and losses, often triggered by infection, fever, impaired oral intake, or excessive insensible losses [1]. In critically ill or elderly patients, such as our 74-year-old woman with ESRD and sepsis, the combination of osmotic stress and hemodynamic instability substantially increases the risk of neurological complications and mortality. Rapid shifts in plasma osmolality are particularly hazardous in comatose or deeply sedated patients, in whom brain adaptive mechanisms may already be exhausted [2]. Therefore, current recommendations emphasize gradual correction of chronic hypernatremia, generally not exceeding 10-12 mmol/L per 24 hours, with close neurological and biochemical monitoring [3].

CRRT offers several advantages over intermittent hemodialysis for managing dysnatremias in unstable patients. Its continuous nature allows sodium and water balance to be adjusted in small, controlled increments, limiting abrupt changes in tonicity. Individualization of the dialysate and replacement-fluid sodium concentration is crucial, especially when baseline serum sodium is far from the standard 140 mmol/L. Practical bedside methods include adding hypertonic saline to increase circuit sodium or sterile water to dilute it, depending on whether hyper- or hyponatremia is present. In our report, Tables 2, 3 (adapted from previously published adjustment schemes [4]) summarize how incremental additions of 3% NaCl or sterile water to a 5-L replacement-fluid bag can fine-tune the final sodium concentration and provide a simple reference for clinicians at the bedside.

**Table 3:** Effect of adding sterile water to a 5-L replacement-fluid bag (baseline Na⁺ 140 mmol/L) Adapted from Baeg et al. [4].

Volume of sterile water added (mL)	Final volume (L)	Final Na⁺ concentration (mmol/L)
0	5	140
250	5.25	133
500	5.5	127
750	5.75	122
1000	6	117
1250	6.25	112

In the present case, we chose CRRT because of the patient’s hemodynamic instability, severe hypernatremia, and need for slow, controlled correction. The decision to add 100 mL of 3% NaCl (≈51 mmol Na⁺) to each 5-L bag was based on mass-balance calculations and published tables, aiming to match the circuit sodium (~150 mmol/L) to the patient’s initial serum sodium. This strategy minimized the transmembrane sodium gradient at the start of therapy and reduced the risk of rapid osmotic shifts. Serial measurements every four to six hours allowed stepwise adjustment of the prescription so that the daily fall in serum sodium remained within the recommended correction limit.

Our patient also illustrates important practical aspects of airway and ventilatory management. As the reviewer correctly noted, a confused mental status alone does not invariably mandate intubation. In this case, however, the patient presented with hypoxemic respiratory failure, impaired airway-protective reflexes, and sepsis-related hemodynamic compromise, which collectively justified early endotracheal intubation to secure the airway and optimize oxygenation and ventilation. Under these conditions, CRRT with individualized sodium prescription, meticulous monitoring, and multidisciplinary collaboration between intensivists and nephrologists enabled safe correction of severe hypernatremia without cerebral edema or osmotic demyelination and facilitated eventual extubation and return to intermittent hemodialysis.

## Conclusions

Severe hypernatremia in critically ill patients with end-stage renal disease is challenging and potentially life-threatening, particularly when it coexists with sepsis and hemodynamic instability. This case highlights that continuous renal replacement therapy can be used not only for solute and fluid control but also as a flexible platform to individualize sodium correction. By adjusting the sodium concentration of the dialysate and replacement solutions with small volumes of 3% NaCl and monitoring serum sodium every four to six hours, we safely reduced the patient’s sodium from 154 to 145 mmol/L over 48 hours without neurological complications.

Simple, bedside tools and tables that link the volume of hypertonic saline or sterile water added to standard CRRT bags with the resulting sodium concentration may help clinicians tailor prescriptions in similar scenarios. Careful planning of the target rate of correction, meticulous biochemical and neurological monitoring, and close collaboration between nephrology and intensive care teams are essential to achieving safe outcomes in patients with severe dysnatremias undergoing CRRT.
